# Bone-eating worms from the Antarctic: the contrasting fate of whale and wood remains on the Southern Ocean seafloor

**DOI:** 10.1098/rspb.2013.1390

**Published:** 2013-10-07

**Authors:** Adrian G. Glover, Helena Wiklund, Sergio Taboada, Conxita Avila, Javier Cristobo, Craig R. Smith, Kirsty M. Kemp, Alan J. Jamieson, Thomas G. Dahlgren

**Affiliations:** 1 Life Sciences Department, The Natural History Museum, Cromwell Road, London SW7 5BD, UK; 2 Depto. de Biología Animal, Facultad de Biología, Universidad de Barcelona, Avda. Diagonal 643, 08028 Barcelona, Spain; 3 Biodiversity Research Institute, Campus Sud, Av. Diagonal 643, 08028 Barcelona, Spain; 4 Centro Oceanográfico de Gijón, Instituto Español de Oceanografía, Avda. Príncipe de Asturias 70 bis, 33212 Gijón, Spain; 5 Department of Oceanography, University of Hawaii at Manoa, 1000 Pope Road, Marine Science Building, Honolulu, HI 96822, USA; 6 Institute of Zoology, Zoological Society of London, Regent's Park, London NV1 4RY, UK; 7 Oceanlab, University of Aberdeen, Institute of Biological and Environmental Sciences, Main St., Newburgh, Aberdeenshire AB41 6AA, UK; 8 Department of Biological and Environmental Sciences, University of Gothenburg, PO Box 463, 405 30 Gothenburg, Sweden; 9 Uni Research, PO Box 7810, 5020 Bergen, Norway

**Keywords:** whale-fall, wood-fall, Annelida, Polychaeta, Siboglinidae, *Xylophaga*

## Abstract

We report the results from the first experimental study of the fate of whale and wood remains on the Antarctic seafloor. Using a baited free-vehicle lander design, we show that whale-falls in the Antarctic are heavily infested by at least two new species of bone-eating worm, *Osedax antarcticus* sp. nov. and *Osedax deceptionensis* sp. nov. In stark contrast, wood remains are remarkably well preserved with the absence of typical wood-eating fauna such as the xylophagainid bivalves. The combined whale-fall and wood-fall experiment provides support to the hypothesis that the Antarctic circumpolar current is a barrier to the larvae of deep-water species that are broadly distributed in other ocean basins. Since humans first started exploring the Antarctic, wood has been deposited on the seafloor in the form of shipwrecks and waste; our data suggest that this anthropogenic wood may be exceptionally well preserved. Alongside the new species descriptions, we conducted a comprehensive phylogenetic analyses of *Osedax*, suggesting the clade is most closely related to the frenulate tubeworms, not the vestimentiferans as previous reported.

## 1. Introduction

A unique characteristic of the Antarctic continent is the complete absence of trees since the Late Eocene, at least 30 million years ago [1]. This has resulted in no significant natural inputs of wood into the marine ecosystem, where the water masses are thought to be isolated by oceanographic features such as the Antarctic circumpolar current (ACC) and Antarctic polar front (APF) [2–4]. By contrast, the Southern Ocean that surrounds Antarctica has some of the highest seasonal abundance of cetaceans anywhere in the world, fuelled by high surface primary productivity and an abundance of krill, *Euphausia superba* [5]. Studies in other ocean basins have suggested that the final resting place of wood and whale remains is often the continental shelf or slope, where they form ephemeral organic-rich ‘island’ habitats for deep-sea fauna to feed from, termed ‘wood-falls’ and ‘whale-falls’ [6–8]. These studies have shown that wood and whale bone are colonized by a remarkable range of specialist deep-sea organisms, the majority of them new to science. Two of the most important in the deep sea are the Xylophagainae bivalves, which bore into wood, and the *Osedax* ‘bone-eating’ worms, members of the annelid clade that bore into vertebrate bones [6,9]. These organisms share remarkable ecological similarities, with their distribution controlled by both dispersal ability and the availability of their respective unique habitats. To date, there have been no experiments conducted on the marine biodegradation of whale bone or wood in the Antarctic, which is a particularly interesting place to test the hypothesis that the ACC or APF is a barrier to deep-water dispersal for *Osedax* and Xylophagainae*.* Furthermore, human activities in Antarctica over the past 100 years have led to a massive reduction in whale populations [10], at the same time as a significant input of wood in the form of shipwrecks and waste [11]. In this paper, we report results from the first experimental study of whale and wood-falls in the Antarctic, testing the effectiveness of dispersal barriers across the Southern Ocean and the potential for the preservation of historical wooden shipwrecks.

For many centuries, humans have been aware of the curious ‘shipworms’ that bore into wood in the marine environment. Sellius [12] published the first significant work in 1733, commissioned by the Dutch to study the animals destroying the wooden pilings protecting the low countries from flooding. He showed that the wood-boring *Teredo* ‘worms’ were in fact extraordinary molluscs. Three centuries of subsequent research has revealed that the Teredinidae shipworms and the closely related Xylophagainae are present in every oxygenated, saline ocean basin so far studied, from the intertidal to the hadal [13,14]. Although there are exceptions, a reasonable generalization is that the Teredinidae are shallow-water specialists on driftwood, littoral wood and man-made wooden structures, with the Xylophagainae specializing on deep-sea sunken wood. Currently, the only seas thought to be free of wood-borers are the Baltic and Black Seas, where well-preserved shipwrecks have been found [15].

It is perhaps unsurprising that the wood-eating ‘shipworm’ molluscs were discovered over 250 years earlier than the bone-eating *Osedax*, given the economic importance of ocean-based wooden structures to humans. Nevertheless, recent experimental and serendipitous discoveries now suggest that the *Osedax* clade may be extremely widespread both geographically and bathymetrically [16–19]. Bone-eating *Osedax* and wood-eating *Xylophaga*, although belonging to different phyla and separated by a vast phylogenetic distance, share remarkable, convergent ecological similarities. Both are specialist biodegraders of hard organic materials, which form spatially and temporally ephemeral ‘island’ habitats on the seafloor. Both use bacterial endosymbionts to potentially feed or bore into their substrates [20–22]. Both *Osedax* and the wood-eating *Xylophaga* can exhibit extreme male dwarfism [23,24], an adaptation to the female's sessile lifestyle. Most significantly, both these types of organism are presumed to have extraordinary powers of dispersal—being able to find the relatively tiny remains of vertebrates, or trees, in the vast expanse of deep-sea sediment [6,16].

To test these powers of dispersal, we conducted an experiment using baited free-vehicle landers at three sites on the Antarctic shelf—two soft-sediment sites at typical shelf depths of 500 m, and an unusual shallow site in the isolated embayment of Deception Island. Based on previous discoveries of differing *Osedax* species in different ocean basins [9,16,18,19], and the apparent abundance of cetaceans (and other marine vertebrates) in Antarctic waters, we hypothesize the presence of *Osedax* but different, locally endemic species to those found before. By contrast, as the Antarctic is free of natural wood sources, and has been for the past 30 myr [25,26], we would not only hypothesize the absence of endemic Antarctic *Xylophaga* species, but also the absence of even temporary invasive *Xylophaga* given the potential power of the ACC or APF as a dispersal barrier [4].

## 2. Methods

**Figure 1.**
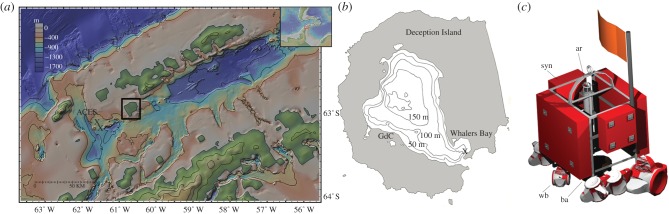
Experiment locations and deployment method. (*a*) Location of ACES landers 1 and 2 in the Bransfield Strait, Antarctica (marked by X); (*b*) location of experiment in Whalers Bay, Deception Island (magnified from black box on (*a*)). GdC, Gabriel de Castilla Antarctic base; (*c*) ACES lander design, height: approximately 1 m. syn, syntactic foam blocks; ar, acoustic release unit; wb, whale bones and wood panels; ba, ballast. (Online version in colour.)

Detailed methods (background to the study areas, settlement substrates, sampling, morphological analysis, molecular analysis including GenBank accession numbers and Primers used) are provided in the electronic supplementary material. In summary, we deployed and recovered two deep-sea landers with approximately 130 kg of wood and whale bone settlement substrates, and a third lander with just whale bone substrate (one vertebra) at three locations on the West Antarctic Peninsula continental shelf, two at approximately 500 m depth in the Bransfield Strait and one at approximately 20 m depth in Whalers Bay, Deception Island (table 1 and figure 1*a*,*b*). The landers were recovered by acoustic release after approximately 1 year on the seafloor and the fauna collected from the substrates for further analysis, including detailed morphology (light, electron microscopy, size measurements) and molecular sequencing and phylogenetic analysis of new species using a combined approach with approximately 1900 bp of nuclear 18S, 600 bp of mitochondrial cytochrome c oxidase I (COI) and 500 bp of 16S genetic markers, with Bayesian phylogenetic analyses and analyses of haplotype networks for COI.

**Table 1.** Experiment locations on the Antarctic shelf.

experiment	package	lat.	long.	depth (m)	implanted	recovered	months on seabed
ACES 1	whale bones, oak and pine planks	63°9.87 S	61°41.34 W	650	3 December 2007	9 February 2009	14
ACES 2	whale bones, oak and pine planks	63°10.98 S	61°38.16 W	568	3 December 2007	9 February 2009	14
Whalers Bay	whale bones	62°59.33 S	60°33.45 W	21	2 January 2009	25 January 2010	12

## 3. Results

### (a) Colonization and biodegradation of wood

**Figure 2.**
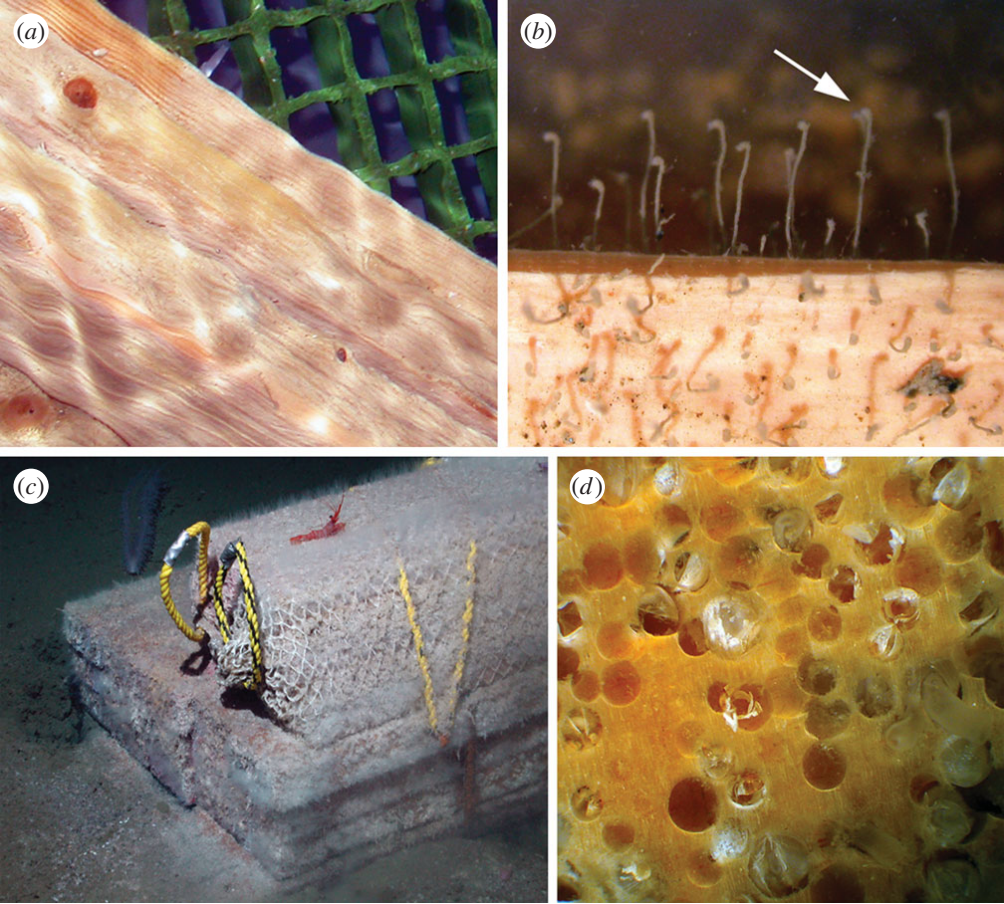
Fate of wood in the deep sea. (*a*) Pine planks recovered from ACES 1 lander in pristine condition after 14 months on seafloor; (*b*) detail of (*a*) showing small Hydrozoa attached to the wood as a hard substrate; (*c*) typical infestation of fir with *Xylophaga* after six months on the seafloor at 1244 m in Santa Catalina Basin, CA, USA; (*d*) inset from (*c*) showing detail of *Xylophaga* borings. (Online version in colour.)

The two landers (ACES 1 and ACES 2; table 1 and figure 1*c*) were successfully recovered on 9 February 2009 from depths of 650 and 568 m, respectively, after 14 months on the seafloor. The recovered wood (pine and oak planks) from both landers was in pristine condition, showing no evidence of macro-boring, no discoloration in portions above the sediment–water interface and no evidence of microbial decay (figure 2*a*,*b*). Examination of the surface of the wood revealed on some pieces numerous unidentified hydroids, attached to the wood and using it as hard substrate, but not boring into it (figure 2*b*). The presence of these hydroids strongly suggests that the wood was not buried in the sediment, which could have prevented it from attack by any locally present or invasive *Xylophaga*. In addition, during over 140 bottom trawls and seafloor photographic surveys associated with the FOODBANCS projects [27], no natural or anthropogenic wood was located on the Antarctic shelf, indicating that wood falls are extremely rare in the region of study.

### (b) Colonization and biodegradation of whale bones

**Figure 3.**
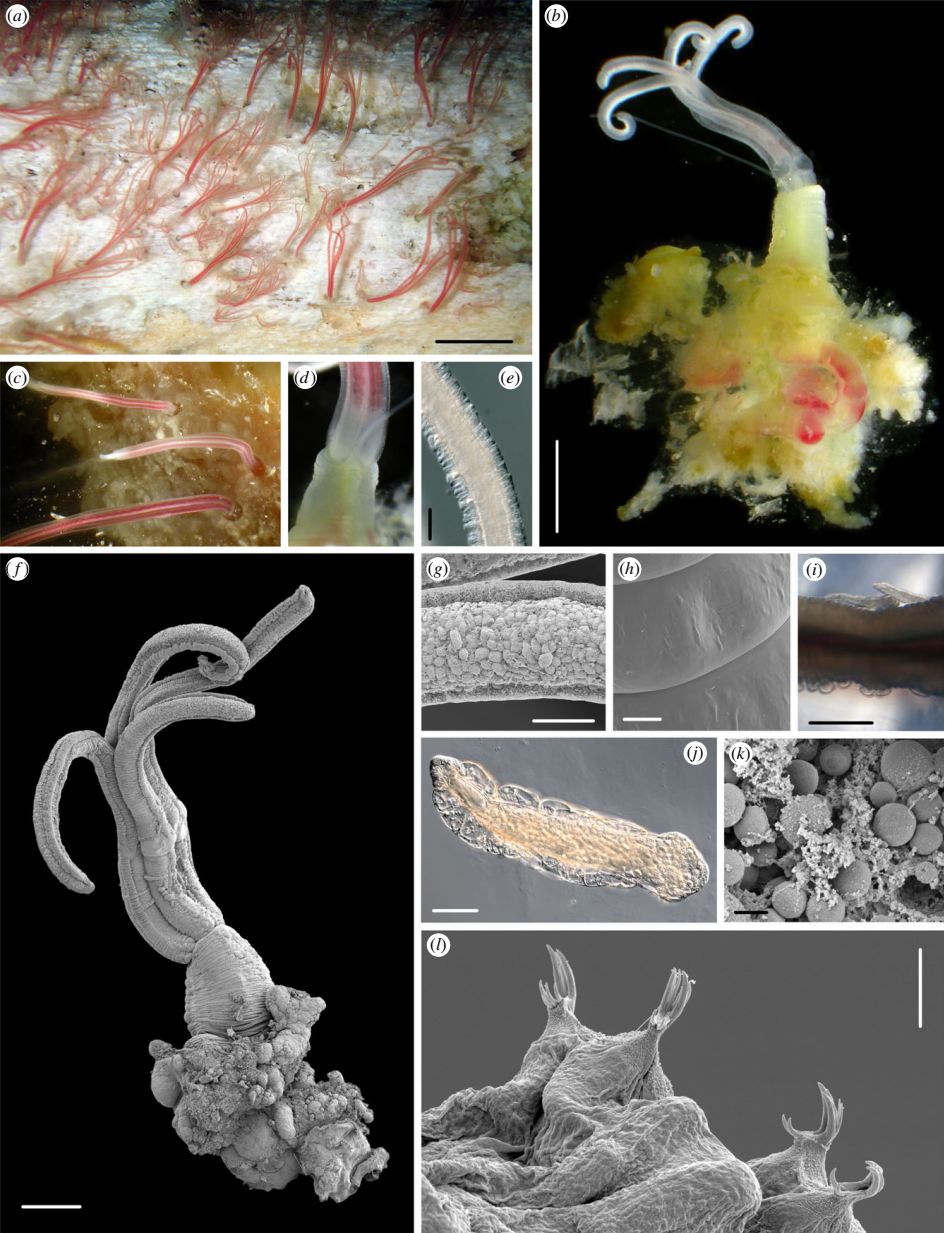
*Osedax antarcticus* sp. nov. (*a*) Live specimens emergent from bone after recovery from the seafloor; (*b*) whole specimen, with palps, oviduct, trunk, root and ovisac, after dissection from bone; (*c*) detail of palps in live specimen; (*d*) detail of collar region; (*e*) light micrograph of palp; (*f*) scanning electron microscope (SEM) of intact specimen; (*g*) SEM detail of palp; (*h*) SEM detail of trunk surface; (*i*) light microscope of two dwarf males attached to trunk (upper side); (*j*) light micrograph of individual male (preserved); (*k*) SEM detail of root transverse section with visible presumed bacteriocytes; (*l*) SEM detail of ophisthosomal chaetae of male. Scale bars (*a*) 1 cm; (*b*) 2 mm; (*e*) 250 µm; (*f*) 500 µm; (*g*) 125 µm; (*h*) 20 µm; (*j*) 100 µm; (*k*) 2 µm; (*l*) 5 µm.

In contrast to the wood samples from the same experimental landers, the pieces of whale bone from the ACES 1 and 2 (500 m depth) experiments were heavily infested with specimens of a new species of *Osedax* ‘bone-eating worm’, described below as *Osedax antarcticus* sp. nov. Every whale bone recovered from both landers was covered in a thick pink-coloured ‘pelt’ of *Osedax* (figure 3*a*). On a single rib bone, a density of 202 specimens per 100 cm^2^ was recorded. The length of the emergent palps was also measured on these live specimens while underwater, with a mean palp length of 10.4 mm (s.d. 5.6 mm) and a maximum palp length in the largest specimen of 25 mm. Observations of the animals on the bone surface (figure 3*a*) were suggestive of two size-classes of animals colonizing the bone, and measurements provided some support for the presence of a size-class peak of individuals with approximately 5 mm palp length and a second peak for individuals with approximately 11 mm long palps (see electronic supplementary material, figure S1). Several other species of annelids were recovered from the ACES bone samples, including several new species of Dorvilleidae (H. Wiklund 2013, unpublished data).

**Figure 4.**
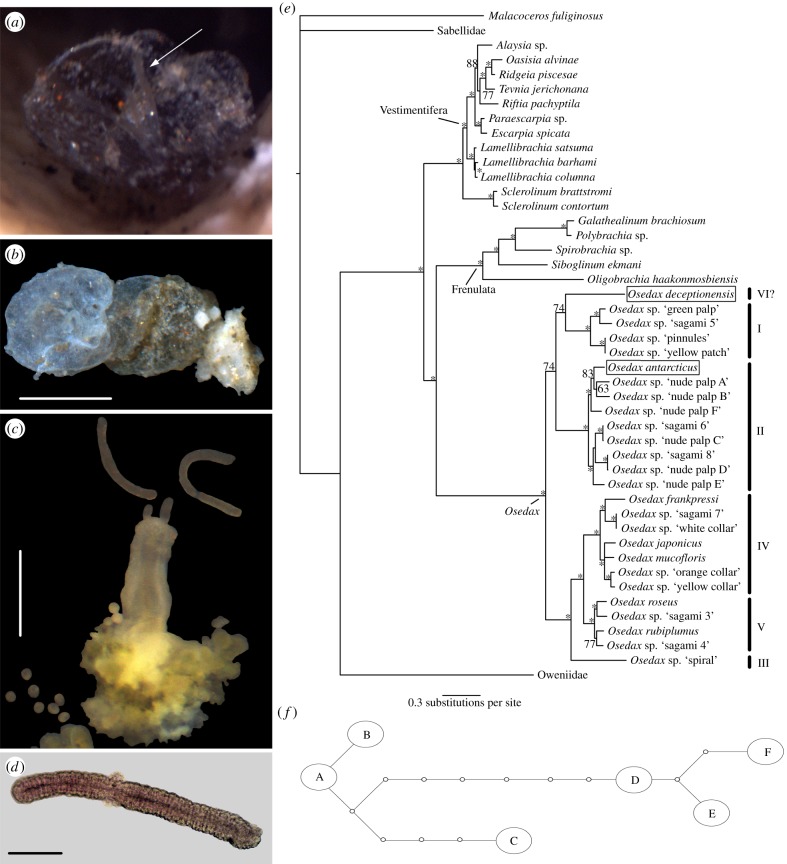
*Osedax deceptionensis* sp. nov and phylogenetic analysis. (*a*) Globular mucous tube of *O. deceptionensis*, emergent from a small hole in the vertebra after recovery from the seafloor, palps arrowed; (*b*) tube after dissection from bone; (*c*) holotype after dissection from bone; (*d*) light microscope detail of palps; (*e*) phylogenetic analysis using Bayesian analysis of all sampled siboglinid polychaetes and *Osedax* including undescribed OTUs on GenBank based on 18S, 16S and COI genetic markers, posterior probability values are indicated, where over 95 marked by an asterisk, species described in this publication boxed; (*f*) *O. antarcticus* sp. nov. COI haplotype network from 12 individuals, haplotype A (*n* = 5), B (*n* = 2), C (*n* = 2), D,E,F (*n* = 1). (Online version in colour.)

The single vertebra recovered at the Whalers Bay mooring was not initially judged to be significantly colonized; however, after several days in an aquarium, a small mucous tube was observed inside a hole in the bone and found to be an extremely small (palp length less than 1 mm) single specimen of *Osedax*, described below as *Osedax deceptionensis* sp. nov. (figure 4). In addition to the *Osedax*, two new species of Dorvilleidae (Annelida) were collected from the aquarium tank associated with the bone [28].

### (c) Systematics

#### (i) Taxonomy

Annelida Lamarck, 1809, Siboglinidae Caullery, 1914.

*Osedax antarcticus* sp. nov. Glover, Wiklund and Dahlgren, 2013 (figure 3*a–l*).

#### (ii) Material examined

*Osedax antarcticus* sp. nov. type material. Bransfield Strait, West Antarctic Peninsula, Southern Ocean, Antarctica collected aboard RV *Laurence M Gould* research cruise LMG-09-02 ‘FOODBANCS2’ project on 9 February 2009 from experimental substrates deployed on 3 December 2007 from RV *Oden* research cruise ‘SWEDARP 07-08’. Holotype: mature adult female (NHM 2013.435), dissected from rib bone collected from ‘ACES 2 Lander’ at 63°10.98’ S 61°38.16’ W deployed on the seafloor in 568 m of water, sample number WW785. Paratypes: mature adult females (NHM 2013.436 and NHM 2013.437), dissected from rib bone of ACES 2 Lander (locality as for holotype) and jaw bones of ACES 1 Lander at 63°9.87’ S 61°41.34’ W deployed on the seafloor in 650 m of water, sample numbers WW791 and WW828. Allotypes: three dwarf males from the tube of paratype NHM 2013.436. Additional non-type material of 36 specimens.

#### (iii) Diagnosis

Voucher specimens (GenBank KF444420, KF444418, KF444422) observed live as four red palps emergent from bone through thin mucous tube (figure 3*a*), length of palps heavily contracted on fixation, measured for formalin-fixed holotype at 2.5 mm, estimated length of palps when fully extended in live specimens 10–15 mm based on observations of a number of individuals. Palps fused for approximately 50% of length, appearance when alive and fused as red wavy stripes, overall appearance on bone in densely colonized regions as a red ‘pelt’ covering bone surface (figure 3*a*,*c*), colour lost on death and fixation. Oviduct similar length to palps, free to base and adjoined to the trunk at collar region, collar region occasionally tinted yellow, varying to entirely yellow trunk (figure 3*b*,*d*). Palps smooth, without pinnules under light microscopy, two approximately 0.05 mm wide ciliary bands running the length of the palp, region between ciliary bands of approximately 0.1 mm filled with region of micropinnules visible under oil-immersion and scanning electron microscope (SEM) giving rugose appearance to the palp surface at high magnification (figure 3*e*–*g*). Smooth trunk region emergent from bone surface, measured in fixed holotype at 0.6 mm width and 1.4 mm length, mean of measured trunk, ethanol-fixed specimens, 0.84 mm width, 1.3 mm length, trunk region without epibiotic bacteria (figure 3*h*), pseudo-segmented ridged appearance under SEM (figure 3*f*,*h*) and with two wide lateral ciliary bands. Roots compact, lobate, heavily vascularized, yellow to green in live specimens, with small lateral projections more heavily pigmented in green–yellow (figure 3*b*), width in holotype 2.7 mm, mean in all specimens measured of 4.4 mm, depth 2.2 mm, but depth possibly unreliable measurement. Root epidermis appearing smooth under low power, under higher magnification rugose with repeating domes of approximately 0.05 mm in width, under high power SEM epidermis with microvilli of approximately 500 nm in length, separated by approximately 500 nm from each other (not illustrated). Exposed root tissue observed under SEM with multiple bacteriocytes, presumed bacteria seen within measuring 0.0015–0.0030 mm (figure 3*k*). Eggs of female rounded, on release from oviduct measuring approximately 0.08 mm. Female tubes consist of thin, mucous sheaths. Mucus tubes extending distally beyond the relaxed animal, most common clear but some opaque white, larger ones branching one or two times.

Male specimens (allotypes) recovered from body wall of paratype NHM 2013.436, length of measured male specimen 0.6 mm, width 0.16 mm (figure 3*j*). Conical prostomium, ciliary band anteriorly (presumed prototroch), body filled with empty sac-like cavities anteriorly, becoming progressively more filled with presumed spermatids towards the posterior opisthosome, where chaetae are located. Chaetae, numbering eight, arranged in two pairs laterally, observed under SEM as prominent raptorial, unhooded hooks emerging from simple conical parapodial lobes, each hook with a set of two to four anteriorly sited teeth and opposing four to six posteriorly sited teeth, giving a grasping appearance (figure 3*l*). Hooks, including parapodial lobe, projecting approximately 0.007 mm from body wall, length and width of hook approximately 0.0038 mm.

#### (iv) Etymology

Pertaining to the type locality, Antarctica.

#### (v) Taxonomy

*Osedax deceptionensis* sp. nov. (Taboada, Cristobo, Avila, Wiklund and Glover, 2013, figure 4*a*–*d*).

#### (vi) Material examined

*Osedax deceptionensis* sp. nov. Type material. Whalers Bay, Deception Island, Bransfield Strait, West Antarctic Peninsula, Southern Ocean, Antarctica collected on 25 January 2010 from experimental whale bone substrate deployed on 2 January 2009. Holotype: mature adult female (BCN CRBA-9621), dissected from vertebra collected at 62°59.33’ S 60°33.45’ W deployed on the seafloor in 21 m of water.

#### (vii) Diagnosis

Holotype (GenBank KF444421, KF444419, KF444428), live specimen as four pale white to translucent palps emerging from a hemispherical mucous tube (figure 4*a*,*b*). Tube in two hemispherical distinct parts: transparent, anterior or water-exposed part; light-brown opaque, posterior or inner-bone part (figure 4*b*). Four smooth palps, without pinnules, detached from the base by the animal after removing it from the bone (figure 4*c*), of equal length approximately 0.6 mm, 0.05 mm wide in ethanol-fixed specimen, under high power light microscopy with slightly rugose appearance (figure 4*d*). No oviduct observed and no trace of any male. Trunk region (0.6 mm long, 0.3 mm wide) in fixed specimen, whitish in live specimens (figure 4*c*). Mouth and gut absent. Lobulated root and ovisac 0.7 mm long, 0.8 mm wide, greenish in live (figure 4*c*). Palps, trunk and ovisac becoming opaque white after preservation. Several spherical eggs (approx. 40) ranging 0.07–0.08 mm diameter, some released from ovisac after specimen dissection (figure 4*c*).

#### (viii) Etymology

Pertaining to the type locality, Deception Island, Antarctica.

#### (ix) Remarks

There are currently five other formally described species in the genus *Osedax*, and a larger number of operational taxonomic units (OTUs) listed as unidentified *Osedax* sp. on GenBank, some of which are mentioned in recent publications (summarized in table S3 of the electronic supplementary material). Of the described species, these are *O. rubiplumus* Rouse *et al*., 2004, *O. frankpressi* Rouse *et al*., 2004, *O. roseus* Rouse *et al*., 2008 described from Monterey Canyon in the northeast Pacific, *O. mucofloris* Glover *et al*., 2005 described from the Swedish North Sea (Skagerrak) and *O. japonicus* Fujikura *et al*., 2006 described from off Kyushu Island, northwest Pacific. *Osedax antarcticus* sp. nov. and *O. deceptionensis* sp. nov. differ clearly from all of the described species in the absence of pinnules on the palps, although we observed some rugose micro-papillated surface under SEM of *O. antarcticus.* They also differ from *O. rubiplumus* and *O. roseus* in that the roots are compact and lobulate rather than long and branched but are similar to *O. frankpressi*, *O. mucofloris* and *O. japonicus* in this regard. *Osedax antarcticus* is further differentiated from *O. japonicus* in the nature of the oviduct, which extends to palp length, but the oviduct was not observed for *O. deceptionensis.* With regard to the OTUs published on the NCBI GenBank, our new species differ genetically from all known species or OTUs of *Osedax* (see section below). *Osedax antarcticus* sp. nov. and *O. deceptionensis* resemble morphologically other OTUs that have been noted to exhibit smooth palps (e.g. *O.* ‘nude-palp A’ [18]) but there is limited morphological detail available for these undescribed species and our species differ genetically. With regard to reproduction, dwarf males (figure 3*j*) were only observed on *O. antarcticus* sp. nov. and counted on seven preserved specimens, with the number of males varying between two and 12 per female, these numbers should be considered a lower limit.

### (d) Genetic barcoding and phylogenetics

We conducted individual alignments and phylogenetic analyses of the annelid family Siboglinidae using the genetic markers 18S, 16S and COI (figure 4*e*), using all known described and OTU *Osedax* taxa that were available. The combined alignment consisted of 3611 characters, of which 18S has 1912 characters, 16S has 574 characters and COI has 1125 characters. The three Bayesian analyses (BA) converged on similar log-likelihood values, mean values for all parameters and clade probabilities; further details of the analyses are provided in the electronic supplementary material. The 50%-majority rule consensus tree from the BA generates 41 nodes, however, only 34 have clade posterior probabilities at 95% or above. There is strong support for the *Osedax* clade, with the Frenulata as its sister group (figure 4*e*). Within *Osedax*, a total of 24 species (including seven described species and 17 OTUs) are now thought to be present (figure 4*e* and electronic supplementary material, table S3), although this may be 23 if we consider OTU ‘sagami 8’ to be the same species as OTU ‘nude-palp D’ with which it differs by only 9 bp within COI and no differences in the other markers. Our evidence supports the synonymy of several other OTUs that are either mentioned in the literature or on GenBank, listed in electronic supplementary material, table S3.

Phylogenetically, we found strong support for the Siboglinidae clades Vestimentifera, *Sclerolinum*, Frenulata and *Osedax*, with support for a sister group relationship between *Osedax* and Frenulata. Within *Osedax*, we found support for five, possibly six, significant clades, labelled in figure 4*e* following the clade numbering system of Vrijenhoek *et al.* [19]. *Osedax deceptionensis* sp. nov. is divergent from all these clades, and we did not confidently resolve its position, it may represent a sixth clade within *Osedax* but this cannot yet be confirmed. In addition, we made an analysis of the haplotype distribution with 12 COI sequences from *O. antarcticus* sp. nov. and six different haplotypes were found (figure 4*f*). Haplotype A consists of five sequences, haplotypes B and C of two sequences each and the others are all single sequences.

## 4. Discussion

The remarkable contrast between our pristine wood blocks and heavily bored whale bones, recovered from the same experimental package at two sites on the Antarctic shelf, is consistent with our hypothesis that *Osedax* are abundant in waters south of the ACC and APF. It confirms that *Osedax* larvae were able to colonize the bone packages after less than one year, whereas Xylophagainae larvae were not. With increasing anthropogenic and potentially natural inputs of wood into the Antarctic, particularly the well-visited West Antarctic Peninsula region, it is important to consider how wood may be biodegraded in the region. Our data cannot be considered conclusive evidence that wood-eating bivalves are absent from the Antarctic. It is possible that our experiments were not deployed for long enough, or that the size of the wood package, or presence of whale bones, inhibited larval settlement. However, lower-latitude deployments of wood panels at similar depths have repeatedly shown heavy infestation by Xylophagainae species after just three months and, in some cases, complete wood destruction after 1 year [6,29–31] (figure 2*c*,*d*).

The most southerly known Xylophagainae (*Xylophaga atlantica* Richards, 1942 and *X. rhyabtshikovi* Kudinova-Pasternak, 1975) were recorded from a shipwreck in 1600 m of water, close to the Falkland Islands at latitude 52° S. By contrast, species of Xylophaginae have been recorded from the high Arctic at latitudes of 72°, suggestive that low temperatures are not a barrier to dispersal for these animals [33]. While the shallow-water teredinid shipworms have an obvious means of long-distance adult dispersal (attached to floating driftwood and wooden ships), the Xylophagainae must rely on larval dispersal in ocean currents, possibly at great depth. Our experiments with wood, although very limited in their geographical scope, do suggest both the absence of locally endemic Xylophagainae populations and the absence of larvae from more broadly distributed species. The observations are suggestive of barriers to the deep-water dispersal of benthic larvae, although the ACC and APF may well be ‘leaky’ over evolutionary timescales, [4]. It is possible that current anthropogenic climate warming may led to increased incursions of larvae into Antarctica as the position of the Polar Front is shifted [34]. The amount of Antarctic marine wood may also increase as a result of human activities such as tourism along the West Antarctic Peninsula, creating a new habitat for invasive species [11]. In addition, our observations will have significance for marine archaeologists interested in the biodegradation of Antarctic shipwrecks such as the Scandinavian pine and oak-built ship *Endurance* used by Ernest Shackleton on his 1914 expedition, now lying on the West Antarctic shelf.

The remarkable colonization of our whale bone experiments by two new species of *Osedax* was in obvious contrast to the untouched wood, but perhaps not entirely unexpected given the abundance of cetaceans in the Southern Ocean [5]. Furthermore, two recent expeditions using remotely operated vehicle have serendipitously discovered intact, natural, whale-falls on the bathyal seafloor in the Bransfield Strait (A. G. Glover 2011, personal observations) and East Scotia Ridge, where abundant but undescribed *Osedax* are reported [35]. Our observations of dense colonization by *O. antarcticus* sp. nov. on the 550–650 m landers are suggestive of an abundant local source of larvae, which may well be the case if natural whale-falls are frequent in the areas. Furthermore, the presence of potentially two size classes of *O. antarcticus* (see electronic supplementary material, figure S1) and the observation of multiple *O. antarcticus* haplotypes at the same lander site (figure 4*f*) are indicative of multiple founder events and an abundant larval source. Antarctic minke whales *Balaenoptera bonaerensis* Burmeister, 1867, humpback whales *Megaptera novaeangliae* (Borowski, 1781), fin whales *Balaenoptera physalus* (Linnaeus, 1758) and blue whales *Balaenoptera musculus* (Linnaeus, 1758) are abundant throughout the Antarctic, and we suspect that the Antarctic holds high *Osedax* diversity and abundance. With regard our experimental method (bones attached to 1 × 1 m landers), we also note that the relatively small size of the experiment compared with an intact whale carcass did not prevent larval entrainment and colonization, and this may well be a useful approach in future studies where such settlement ‘traps’ could be deployed to study deep-sea dispersal in particular target taxa, such as *Osedax*.

*Osedax deceptionensis* sp. nov. is remarkable in that it is recorded from the shallowest depths so far for the genus, extending the range for *Osedax* from 2893 [9] to just 21 m in this study. The locality of its discovery is also of interest: an extremely isolated drowned (and still active) caldera with a maximum depth of just 180 m and a shallow sill with limited water exchange to the open sea [36]. However, whale bones are not a rarity in the caldera of Deception Island. Indeed, they occur widely on the beach and in the shallow subtidal—relics from the early-twentieth century whaling industry (see electronic supplementary material). It is an interesting question as to whether *O. deceptionensis* sp. nov. is mainly using anthropogenic, ‘dumped’ whale bones as a food source, or is reliant on a natural input of fresh whale carcasses. Natural bones will differ in that the historical bones were often boiled (for oil extraction) prior to dumping, although we now believe that *Osedax* has a general preference for bones with lower oil content [37]. There is little evidence either way, but given that there have been no observations of *Osedax* on the shallow subtidal historical bones, as yet, we may assume for the moment that the species is reliant on the more organic-rich fresh bones as that is the only habitat it has been recorded on. Given the periodic disturbances in the form of eruptions, the deposition of volcanic and fluvial sediments [38] and other effects such as the mechanical abrasion in the littoral zone, it may well be that periodic recolonization is required from outside the island. Further collecting in the region will provide more clues to this interesting puzzle, that may well link the current distribution of bone-eating worms with an unusually intense human impact in the area.

Morphologically, the two new species of *Osedax* described here are different to other described species in the form of the ‘smooth’ palps, without the complex pinnule structure seen on other *Osedax*. This could be construed as an adaptation to the relatively high oxygen content of the cold Antarctic waters; however, smooth palps have been informally observed on a number of undescribed *Osedax* species from lower latitudes [19]. One characteristic feature of *Osedax* is the presence of dwarf males; we did not observe any on the single, tiny specimen of *O. deceptionensis* sp. nov. but they were seen on several specimens of *O. antarcticus* sp. nov. (figure 3*j*). The chaetae of the males, observed in high-resolution SEM (figure 3*l*), show a remarkable opposition of two sets of teeth (rostral and subrostral teeth), and we hypothesize that they are adapted to grasping the females, or bone, on settlement.

We performed a phylogenetic analysis of the annelid family Siboglinidae and here include the full range of available siboglinid sequences alongside the 24 (or 23) suggested *Osedax* OTUs (figure 4*e* and electronic supplementary material, table S3). At higher levels, we find strong support for the traditional clades Vestimentifera, *Sclerolinum*, Frenulata and the relatively new clade *Osedax*. We support the proposition of Rouse [39] to maintain use of these names within Siboglinidae, without giving them rank, although we elect to not use the name Monilifera *sensu* Rouse [39] to refer to both Vestimentifera and *Sclerolinum*. Our analysis of the phylogenetics of *Osedax* differs from the two previous studies [9,16] in that we resolve Frenulata, the predominantly mud-dwelling group formerly referred to as pogonophorans, as the *Osedax* sister group, rather than as Vestimentifera and *Sclerolinum*. This is based on a combined analysis of three genes, rather than the two of the previous studies. It is interesting to speculate on the evolutionary origin of the remarkable *Osedax* worms, but there can be no doubt that the data are still extremely limited. If anything, we can hypothesize that they are more closely related to the sediment-dwelling chemosynthetic siboglinids (Frenulata), the ancestors of which were perhaps able to colonize whale bones in soft-sediment environments. A problem in this analysis is the very limited genetic sampling of Frenulata, which is the most speciose siboglinid group, but the least sampled [40].

Within *Osedax*, we also find strong support for the five clades noted by Vrijenhoek *et al* [19], although *O. deceptionensis* sp. nov. was highly divergent and may represent a sixth clade. *Osedax antarcticus* fell within a clade (II) of undescribed OTUs that have been observed to also lack pinnules on their palps. Although there is rather little morphological or habitat data available for many of the undescribed OTUs (see electronic supplementary material, table S3), it is striking that there is no clear geographical or bathymetric pattern among the *Osedax* clades. With the exception of the monospecific clades III and VI, the clades all contain species that have been recorded from multiple ocean basins and depth ranges of a minimum of 1500 m (see electronic supplementary material, table S3). The two new Antarctic species are not sister-taxa, as might be expected if *Osedax* had colonized the Antarctic once, early in its evolution. There are, in essence, no indications of vicariance-driven phylogeographic or phylobathymetric patterns in *Osedax*. We must therefore speculate down different routes as to what has driven speciation in *Osedax*. This might include habitat specialization that is independent of geography or bathymetry; in this regard, the distribution of the great whales is probably concordant in that different species of whale-fall are likely to be very broadly distributed. A single whale-fall may exhibit a range of microhabitats delicately separated in space and time offering a basis for speciation [41–43]. Another, perhaps more controversial suggestion is that the *Osedax* larvae are so prevalent, and the species so mixed, that the successful colonization of a carcass may come down to a combination of chance and interspecific competition during the initial growth phases. We may not know the answer to these questions without a greater understanding of siboglinid larval distribution, settlement and growth.

With increased sampling, the growing diversity and distribution of *Osedax* is becoming all the more remarkable given that new species of the group are being discovered in even very shallow depths, close to marine biological stations that have been active for many decades (e.g. Deception Island and the Swedish West Coast). Based on the simple premise that almost every whale-fall so far sampled has revealed new species, the global diversity of this group may be very high, far exceeding the current 24 known OTUs. *Osedax* and the ecologically similar Xylophagainae bivalves are the ecosystem engineers of organic hard substrates, recycling the organic material trapped in refractory bone and wood matrices through specialized adaptations and symbiotic bacteria. In the Antarctic, a unique place in so many respects, our simple experiment has shown how the dispersal of these species controls the very contrasting fate of wood and whale remains on the Southern Ocean seafloor.
